# Abnormally Large Baseline P300 Amplitude Is Associated With Conversion to Psychosis in Clinical High Risk Individuals With a History of Autism: A Pilot Study

**DOI:** 10.3389/fpsyt.2021.591127

**Published:** 2021-02-09

**Authors:** Jennifer H. Foss-Feig, Sylvia B. Guillory, Brian J. Roach, Eva Velthorst, Holly Hamilton, Peter Bachman, Aysenil Belger, Ricardo Carrion, Erica Duncan, Jason Johannesen, Gregory A. Light, Margaret Niznikiewicz, Jean M. Addington, Kristin S. Cadenhead, Tyrone D. Cannon, Barbara Cornblatt, Thomas McGlashan, Diana Perkins, Larry J. Seidman, William S. Stone, Ming Tsuang, Elaine F. Walker, Scott Woods, Carrie E. Bearden, Daniel H. Mathalon

**Affiliations:** ^1^Department of Psychiatry and Seaver Autism Center for Research and Treatment, Icahn School of Medicine at Mount Sinai, New York, NY, United States; ^2^San Francisco VA Health Care System, San Francisco, CA, United States; ^3^Department of Psychiatry, University of California, San Francisco, San Francisco, CA, United States; ^4^Department of Psychiatry, University of Pittsburgh, Pittsburgh, PA, United States; ^5^Department of Psychiatry, University of North Carolina, Chapel Hill, NC, United States; ^6^Department of Psychiatry, Zucker Hillside Hospital, New York, NY, United States; ^7^Departments of Psychology and Psychiatry, Atlanta VA Health Care System and Emory University, Decatur, GA, United States; ^8^Departments of Psychology and Psychiatry, Yale University, New Haven, CT, United States; ^9^Department of Psychiatry, University of California, San Diego, San Diego, CA, United States; ^10^Department of Psychiatry, Harvard University, Cambridge, MA, United States; ^11^Department of Psychiatry, University of Calgary, Calgary, AB, Canada; ^12^Departments of Psychiatry and Biobehavioral Sciences and Psychology, University of California, Los Angeles, Los Angeles, CA, United States

**Keywords:** autism spectrum disorder, psychosis, P300, EEG, conversion, prodrome

## Abstract

Psychosis rates in autism spectrum disorder (ASD) are 5–35% higher than in the general population. The overlap in sensory and attentional processing abnormalities highlights the possibility of related neurobiological substrates. Previous research has shown that several electroencephalography (EEG)-derived event-related potential (ERP) components that are abnormal in schizophrenia, including P300, are also abnormal in individuals at Clinical High Risk (CHR) for psychosis and predict conversion to psychosis. Yet, it is unclear whether P300 is similarly sensitive to psychosis risk in help-seeking CHR individuals with ASD history. In this exploratory study, we leveraged data from the North American Prodrome Longitudinal Study (NAPLS2) to probe for the first time EEG markers of longitudinal psychosis profiles in ASD. Specifically, we investigated the P300 ERP component and its sensitivity to psychosis conversion across CHR groups with (ASD+) and without (ASD–) comorbid ASD. Baseline EEG data were analyzed from 304 CHR patients (14 ASD+; 290 ASD–) from the NAPLS2 cohort who were followed longitudinally over two years. We examined P300 amplitude to infrequent Target (10%; P3b) and Novel distractor (10%; P3a) stimuli from visual and auditory oddball tasks. Whereas P300 amplitude attenuation is typically characteristic of CHR and predictive of conversion to psychosis in non-ASD sample, in our sample, history of ASD moderated this relationship such that, in CHR/ASD+ individuals, enhanced – rather than attenuated - visual P300 (regardless of stimulus type) was associated with psychosis conversion. This pattern was also seen for auditory P3b amplitude to Target stimuli. Though drawn from a small sample of CHR individuals with ASD, these preliminary results point to a paradoxical effect, wherein those with both CHR and ASD history who go on to develop psychosis have a unique pattern of enhanced neural response during attention orienting to both visual and target stimuli. Such a pattern stands out from the usual finding of P300 amplitude reductions predicting psychosis in non-ASD CHR populations and warrants follow up in larger scale, targeted, longitudinal studies of those with ASD at clinical high risk for psychosis.

## Introduction

While autism spectrum disorder (ASD) and the schizophrenia spectrum disorders (SCZ) are considered diagnostically distinct, they share phenotypic features, genetic overlap, and a common historical background (1, 2) that highlight the possibility of related neurobiological substrates. As a neurodevelopmental disorder, ASD diagnosis –characterized by impaired social interaction and communication, alongside repetitive and restricted behaviors and interests (3) occurs in early childhood. SCZ is also characterized by impairments in social interactions, but hallmark symptoms of delusions, hallucinations, disorganized thought and behavior, and a constellation of negative symptoms, typically emerge in late adolescence and early adulthood (4). Yet, epidemiological studies also point to considerable overlap between the two disorders. Estimates of SCZ rates in ASD, for example, are 5–35% higher than in the general population (5–7), while rates of ASD diagnoses in SCZ patients range from <1–52% (8). Importantly, prodromal symptoms of SCZ that precede full-blown illness also include social deficits (9) that share some overlap with core features of ASD. In addition, cognitive deficits are pervasive in both ASD and SCZ, as well as in prodromal SCZ. Indeed, tasks that probe attention, memory, and executive functioning find differences in processing speed, accuracy, and perceptual discrimination/detection thresholds compared to typically developing (TD) control cohorts across disorders (10–12).

Recent trends in the schizophrenia field have focused on examining clinical and neurobiological characteristics in individuals at clinical high risk for psychosis (CHR) in order to identify which features are most predictive of transition to full-blown psychosis. Clinically, some of the best predictors of conversion to psychosis include genetic risk, history of substance abuse, and severity of social impairment (13). Neurologically and neuropsychologically, brain volume abnormalities (14, 15), reduced processing speeds and worse verbal memory (16) are associated with increased risk and an earlier psychosis conversion in CHR individuals. Until recently, it was unknown whether individuals with ASD who presented at CHR services showed similar prodromal features and conversion rates to those seen in the broader CHR general population. However, a recent study from the second wave of the North American Prodrome Longitudinal Study (NAPLS2) revealed that CHR individuals with prior ASD diagnoses had more social impairment than other CHR individuals, but similar positive symptoms of psychosis and similar rates of converting to co-morbid psychotic illness (17). However, it is not yet known whether neurological profiles and predictors of conversion to psychosis are similar between CHR individuals with and without ASD.

Event-related potentials (ERPs) have been widely used in understanding altered information processing in clinical vs. non-clinical populations. In SCZ, reduced P300 amplitude during detection of an infrequent target stimulus is among the most reliable and replicable findings (18–20). P300 is a positive-going ERP associated with shifting and allocation of attention, as well as stimulus salience (21–27), where larger amplitudes reflect larger resource allocation toward these processes. The robust amplitude reduction in SCZ suggest that the P300 might be a possible biomarker for the illness (28). Moreover, attenuated P300 is also seen in CHR individuals (29, 30) and may be useful as a predictive tool when identifying individuals at risk for psychosis conversion (31–33) and considering preventative interventions.

P300 can be divided into two subcomponents: P3a and P3b. P3a is maximal over frontocentral scalp and reflects attention orienting toward novel stimuli that are not behaviorally-relevant, in other words, distractors requiring no response (34–38). P3b, on the other hand, is maximal over central-parietal scalp and reflects allocation of attention toward infrequent stimuli that require behavioral response. In schizophrenia, P3b amplitude deficits are well-replicated, particularly in the auditory modality (18, 19, 39–43). Auditory P3a amplitude deficits have also been detected (19, 29, 42, 44–50), though they may be less robust (44, 51, 52). In CHR, both P3a and P3b amplitude reductions have been identified (29, 46, 50, 53–60), with emerging evidence that auditory P3b amplitude may be predictive of conversion to psychosis (33, 60).

The P300 literature in ASD is less clear than in SCZ and CHR. A recent meta-analysis of the P3a and P3b found only reduction in P3b amplitude to be a reliable alteration, whereas P3b latency and both P3a amplitude and latencies were generally similar to controls (61). Clear differences in P300 response to auditory vs. visual stimuli have not been reported, though in general there are more systematic findings of impaired auditory processing and enhanced visual perceptual functioning in the ASD literature broadly. Whether there is a particular pattern of P300 alterations that characterizes individuals with ASD and CHR or predicts who in this population will develop full-blown psychotic illness has not been examined.

The present study leveraged a large, longitudinal study of CHR individuals to examine whether the neural profile and predictors of conversion to psychosis are comparable between individuals with and without co-morbid ASD. In particular, we focused on early attention-modulated indices in response to both attended (P3b) and task-irrelevant (P3a) sensory input. By testing both auditory and visual sensory modalities, we further examined whether, as in CHR more generally, sensory domain affects the predictive utility of brain-based measures dependent on ASD status. We hypothesized that whereas consistent P300 amplitude attenuations are predictive of conversion to psychosis in general CHR populations, P3b deficits may be more specific in those with ASD history and visual P300 deficits may be lacking regardless of conversion. Because there have been no longitudinal studies of neural markers of psychosis risk and development in ASD, this study capitalized on a large-scale study in order to identify a rare subset of individuals with both ASD and CHR. Though our sample size is small and our findings preliminary, this exploratory work offers the first window into brain-based predictors of psychosis conversion in individuals with ASD and a launching point for future, larger studies.

## Method

### Participants

EEG data were available from the baseline visits of 304 patients who participated in the North American Prodrome Longitudinal Study (NAPLS2) (62), a consortium of eight research centers studying CHR between 2009 and 2013, comprising help-seeking individuals ages 12–35 years, observed for up to 2½ years. These patients represent a subset of the full NAPLS2 cohort who completed both the auditory and visual oddball tasks (see below) at baseline and either converted to psychosis anytime within the 24-mo follow-up period or were followed through to the 24-month visit without converting. All CHR individuals met one or more of the three Criteria of Prodromal Syndromes (COPS): attenuated positive symptom syndrome (APSS), genetic risk and deterioration (GRD), and/or brief intermittent psychotic syndrome (BIPS). APSS requires at least one attenuated positive psychotic symptom, begun or worsened in the past year, and of insufficient severity to meet diagnostic criteria for a psychotic disorder. GRD is defined in NAPLS2 as a combination of functional decline (30% or greater drop in Global Assessment of Function score over the month preceding the baseline visit, as compared to 12 months prior) and genetic risk, defined as either schizotypal personality disorder or a first-degree relative with a schizophrenia spectrum disorder. BIPS reflects the presence of a one or more positive psychotic symptom meeting severity threshold but too brief to meet diagnostic criteria for psychosis (63). There was no formal testing or screening for peripheral sensory deficits as part of study procedures or exclusion criteria.

For this study, CHR participants were grouped based whether or not they had a comorbid ASD diagnosis noted at baseline (ASD+: comorbid ASD; ASD–: no ASD) to predict whether they converted to psychosis (Conv+: converter; Conv–: non-converter) within the 2 years following their baseline visit. All patients in the ASD+ group met DSM-IV criteria for Autistic Disorder, Asperger's Disorder, or Pervasive Developmental Disorder-Not Otherwise Specified (PDD-NOS) using a combination of DSM-IV checklist during baseline clinical interview, medical records, and caregiver report of historical diagnosis. All patients designated as Conv+ experienced conversion from CHR state to psychosis, determined by meeting the Structured Interview for Psychosis-Risk Syndromes (SIPS) (64, 65), Presence of Psychotic Symptoms criteria (13). Conversion decisions were discussed and approved on a weekly consensus call. In total, of the 304 participants with included data, 290 did not have ASD (ASD–) and 14 had previous ASD history (ASD+). Within the ASD– group, 71 converted to psychosis (Conv+); conversion to psychosis occurred in four participants within in the ASD+ group. Table 1 summarizes demographic information and assessment scores by group. The sample yielded closely age-matched groups (Main Effect Conversion: F_1,300_ = 0.61, *p* = 0.44; Main Effect ASD: F_1,300_ = 3.69, *p* = 0.056; Conversion × ASD Interaction: F_1,300_ = 0.042, *p* = 0.84). Illness level also did not differ among groups at baseline. In particular, across SIPS positive, negative, disorganization, and general subscales, there were no main effects of conversion status (Positive: F_1,298_ = 0.60, *p* = 0.44; Negative: F_1,300_ = 0.24, *p* = 0.68; Disorganization: F_1,299_ = 0.037, *p* = 0.85; General: F_1,298_ = 0.24, *p* = 0.88) or ASD diagnosis (Positive: F_1,300_ = 0.14, *p* = 0.71; Negative: F_1,298_ = 0.017, *p* = 0.90; Disorganization: F_1,299_ = 0.45, *p* = 0.50; General: F_1,298_ = 0.48, *p* = 0.49), and no significant interaction effects between conversion status and ASD (Positive: F_1,300_ = 1.96, *p* = 0.16; Negative: F_1,298_ = 0.97, *p* = 0.22; Disorganization: F_1,299_ = 1.48, *p* = 0.23; General: F_1,298_ = 0.072, *p* = 0.79) (see Table 1).

**Table 1:** Participant demographics.

				**Mean SOPS scores (SD)**			
**Group**	**N**	**Age (SD)**	**Females (%)**	**Positive**	**Negative**	**Disorganized**	**General**
Conv–/ASD– (non-converter)	219	19.56 (4.63)	101 (46.11)	11.60 (4.23)	11.71 (6.14)	4.87 (3.23)	8.96 (4.29)
Conv+/ASD– (converter)	71	18.79 (3.67)	29 (40.85)	13.54 (3.84)	12.10 (6.48)	6.31 (3.75)	9.52 (4.40)
Conv–/ASD+ (non-converter)	10	17.28 (2.91)	1 (10)	13.80 (3.23)	13.80 (4.49)	6.80 (2.62)	8.40 (5.70)
Conv+/ASD+ (converter)	4	15.98 (2.56)	0 (0)	12.25 (3.10)	10.50 (5.51)	5.75 (5.50)	8.25 (0.96)

The Institutional Review Boards of the eight participating sites approved all study protocols. All adult subjects gave informed consent. Minor subjects provided verbal assent while their parents/guardians provided written informed consent.

### Oddball Paradigm

The experiment consisted of two (visual, auditory) three-stimulus oddball paradigms, where in addition to the frequent, standard stimulus and the rare, target stimulus, there were also rare novel, task-irrelevant stimuli (37). Each oddball task (i.e., visual and auditory) comprised three blocks of 150 trials, of which 80% of trials were standards (visual: small blue circle presented at the vertical and horizontal meridian; auditory: 500 Hz, 50 ms tone with a 5 ms rise/fall time at 62 dB). An additional 10% of trials were target stimuli (visual: large blue circle presented at the vertical and horizontal meridian; auditory: 1,000 Hz, 50 ms tone with a 5 ms rise/fall time at 62 dB), and 10% were novel stimuli (visual: fractal images; auditory: man-made and natural sounds) that were, on average, 250 ms in duration and presented at 62 dB (66). All visual stimuli were presented for 500 ms and the difference in radius between the target and standard circle was ~104:67 in ratio. Stimuli were presented in the same pseudorandom order for all participants. Target and novel stimuli were not allowed to repeat in a sequence such that two deviant stimuli could not occur in a row.

Participants were instructed to respond to the target stimulus and withhold a response to both standard and novel stimuli. Participants indicated their response by pressing a button on a Cedrus® response box using the index finger of their dominant hand. Incorrect trials were excluded from EEG analysis. There was a fixed, 1,250 ms stimulus onset asynchrony (SOA) between auditory oddball trials such that each block lasted approximately 3 min. Visual oddball trials were jittered between 1,500 and 2,500 ms (mean SOA = 2 s) to avoid simultaneous presentation with auditory stimuli from a background mismatch negativity task. Stimulus presentation was implemented with Presentation® software (Version 13.0, Neurobehavioral Systems, Inc., Berkeley, CA, www.neurobs.com).

### Electroencephalographic Data Acquisition and Pre-processing

Participants sat in front of a computer monitor with a screen resolution of 1,024 × 768 and a refresh rate of 60 Hz. As described in (32), EEG was recorded at 1024 Hz using either a 32-channel (4 NAPLS2 sites) or 64-channel (remaining 4 sites) BioSemi ActiveTwo recording system (BioSemi, Amsterdam, Netherlands). Additional electrodes were placed on the nose and mastoids with vertical electrooculogram (VEOG) recorded at electrodes placed above and below the right eye and horizontal (HEOG) electrodes at the outer canthus of each eye.

Continuous EEG data were re-referenced to averaged mastoids and high-pass filtered (0.1 Hz). Data were then processed using a modified version [see (32) for detail] of the Fully Automated Statistical Thresholding for EEG artifact Rejection (FASTER) Routine (67), with additional modification of ICA component selection as per previous literature [see (68)] to ensure proper removal of visual artifacts in the visual oddball task where blinks and saccades may be temporally correlated with ERP components. Continuous EEG data were segmented from −1,000 to 2,000 ms time-locked to the onset of the stimulus during FASTER pre-processing. Last, ERP data were baseline corrected (−100 to 0 ms) and low-passed filtered at 30 Hz.

### Statistical Analysis

The measure of interest in the oddball task was P300 amplitude, which was disambiguated by computing difference waveforms by subtracting the standard ERP form target (P3b) and novel (P3a) ERPs separately for the auditory and visual tasks. P300 amplitude was defined based on previous literature (32, 69) as the peak amplitude, elicited between 235 and 400 ms follow stimulus onset for auditory stimuli and 230–500 ms for visual stimuli. Peak amplitude was identified within each of these windows at Cz for P3a (in response to task-irrelevant, non-target stimuli) and at Pz for P3b (in response to target stimuli), based on previous literature showing these sites are where P3a and P3b, respectively, have maximal amplitude. Average amplitude value within a 30 ms window centered around this peak was extracted. Thereafter, a statistical correction was applied to all ERP measures to adjust for normal aging effects and data collection site (32). In short, the age-corrected P300 amplitude z-score describes the amount, in standard units, that a participant's amplitude deviates from the value expected for a healthy individual of a given age assessed at a specific consortium site (70–72).

A binomial logistic regression model was applied to examine whether the relationship between baseline P300 amplitude and later psychosis conversion status (Conv+, Conv–) was moderated by whether or not individuals had a prior ASD diagnosis. The effects of interest were the main effect of P300 amplitude and the interaction term between ASD and P300 amplitude. Separate models were used for the four conditions (modality: auditory, visual; stimulus type: target, novel) to prevent collinearity of predictors. Main effects of amplitude would replicate prior findings showing that P300 predicts conversion to psychosis in CHR samples. A statistically significant ASD × P300 amplitude interaction would suggest the association between P300 amplitude and converting to psychosis changes based on ASD status. The ASD+ group was used as the reference condition, and bootstrapping procedures with 1,000 resamples were used to assess statistical significance and model stability. When significant interactions between P300 amplitude and ASD diagnosis were present, follow up analyses with simple slopes were computed to better understand the moderating effect of ASD.

To ensure any neural differences detected weren't simply downstream effects of differing behavioral performance across participants, behavioral measures of accuracy and reaction time of target detection were analyzed separately using a 3-way [ASD diagnosis (ASD+, ASD–) × Conversion status (Conv+, Conv–) × Modality (auditory, visual)] repeated measures analysis of variance (ANOVA). Accuracy was calculated as percent correct and comprised total hits [i.e., responding to (70, 71) the target] and correct rejections (i.e., withholding a response to novel, task-irrelevant stimuli and frequent standards) given as the following formula: $\frac{Rejections_{standard}+Rejections_{novel}+Hits_{target}}{All_{standard}+All_{novel}+ALL_{target}}$. Reaction time reflected the time taken to press the button (i.e., respond) to Target stimuli in each modality.

Significance testing was conducted with an alpha level of *p* = 0.05, with *p*-values generated from bootstrapping with 1,000 resamples. Since this study was designed to be hypothesis-generating given the small number of individuals with ASD history in the NAPLS2 cohort, we did not correct for multiple comparisons in order to reduce the chance of type two error.

## Results

### Oddball Behavioral Data

Behavioral performance is summarized in Table 2. The analysis of response accuracy revealed no significant main effects or interactions between ASD and conversion status, and all participants were highly accurate. Results indicated that, across groups, participants were equally accurate on the visual [(0.99 ± 0.008), (Mean ± SE)] as they were on the auditory (0.98 ± 0.006) oddball task (F_1,300_ = 0.26, *p* = 0.61). There were no differences in accuracy between groups based on Conversion status (F_1,300_ = 0.65, *p* = 0.646; Conv–: 0.99 ± 0.006; Conv+: 0.98 ± 0.009) or ASD diagnosis (F_1,300_ = 0.10, *p* = 0.75; ASD–: 0.98 ± 0.002; ASD+: 0.99 ± 0.01). Analysis of reaction time also showed no significant main or interaction effects (see Table 2). Across conversion status (Conv–: 486.35 ± 13.70 ms; Conv+: 483.94 ± 21.78 ms), ASD diagnosis (ASD–: 501.51 ± 5.78 ms; ASD+: 468.78 ± 25.07 ms), and modality (Auditory: 485.15 ± 15.25 ms; Visual: 485.15 ± 13.50 ms), groups were comparable in their reaction time.

**Table 2:** Oddball behavioral data ANOVA summary.

	**df**	**F**	***p***	**η_p_^2^**
**Accuracy**				
Modality	1,300	0.26	0.61	0.001
Conversion status	1,300	0.21	0.65	0.001
ASD status	1,300	0.10	0.75	<0.001
Modality × Conversion	1,300	0.87	0.35	0.003
Modality × ASD	1,300	0.047	0.83	<0.001
Conversion × ASD	1,300	0.14	0.71	<0.001
Conversion × ASD × Modality	1,300	0.028	0.87	<0.001
**Reaction time**				
Modality	1,300	<0.001	1.00	<0.001
Conversion status	1,300	0.009	0.93	<0.001
ASD status	1,300	1.62	0.93	<0.001
Modality × Conversion	1,300	0.88	0.35	0.003
Modality × ASD	1,300	0.44	0.51	0.001
Conversion × ASD	1,300	2.83	0.20	0.005
Conversion × ASD × Modality	1,300	0.46	0.83	<0.001

### ERP Data

There were no significant differences in the number of included ERP trials between groups, overall or as a function of stimulus type or modality (Conversion: F_1,300_ = 0.52, *p* = 0.47; ASD: F_1,300_ = 0.007, *p* = 0.94; Conversion × ASD: F_1,300_ = 0.63, *p* = 0.63; 2-way interactions with Conversion: *p* > 0.05; 2-way interactions with ASD: *p* > 0.05; 3- and 4-way interaction with Conversion and ASD status: *p* > 0.05).

**Figure 1:**
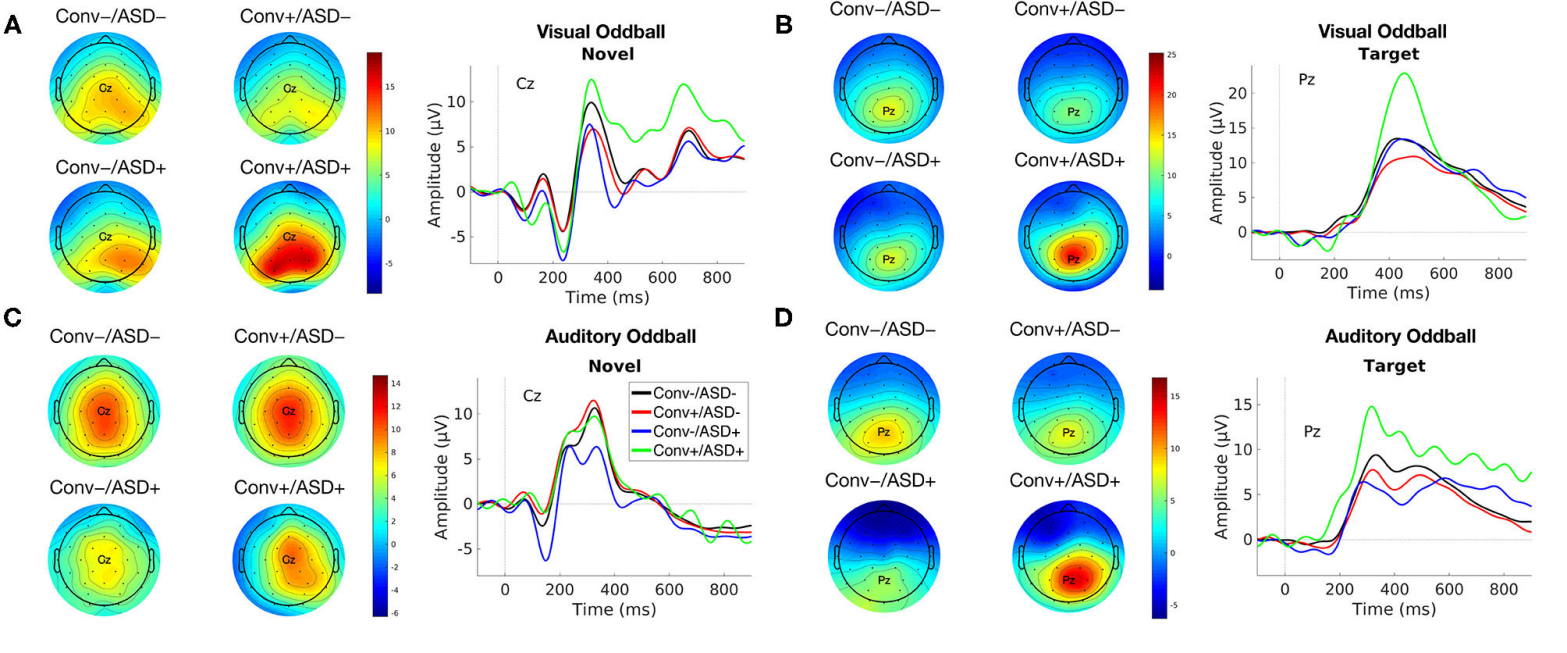
Group average ERP waveforms for modality [Visual **(A,B)** and Auditory **(C,D)**] and Stimulus Type [Novel **(A,C)** and Target **(B,D)**] for Conv–/ASD– (black), Conv–/ASD+ (blue), Conv+/ASD– (red), and Conv+/ASD+ (green). Stimulus onset occurred at 0 ms. Scalp topographic maps of the mean P3a/P3b amplitude in response to the Target/Novel stimulus type around the mean latency ± 10 ms.

**Figure 2:**
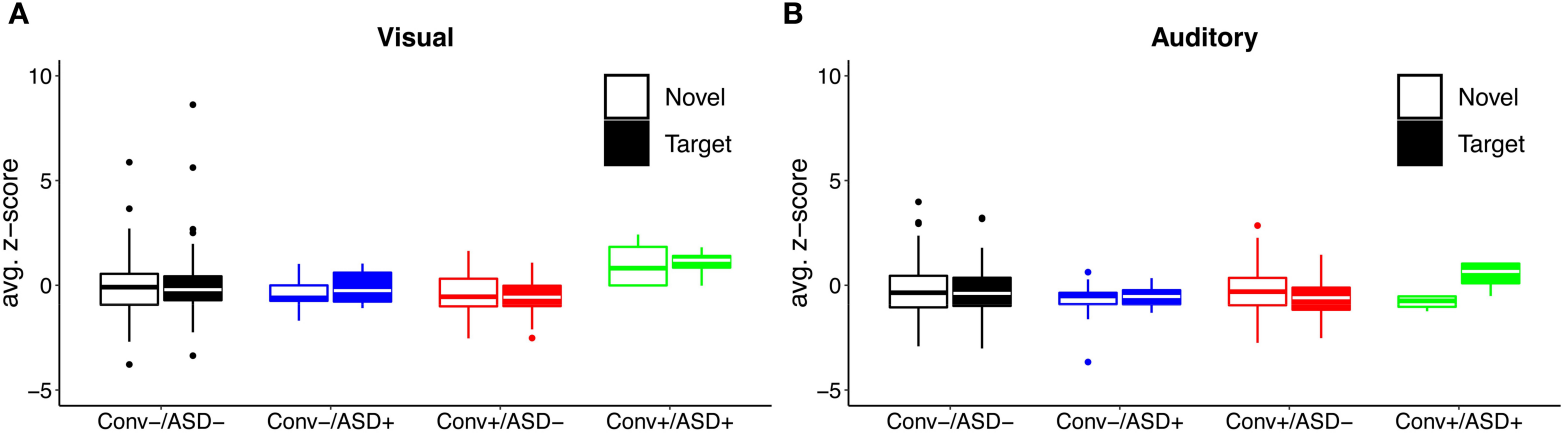
Distribution of **(A)** visual and **(B)** auditory P300 amplitude as a function of ASD history and conversion status.

Our central question was whether the predictive relationship between P300 amplitude and conversion status was moderated by history of ASD diagnosis. Figure 1 shows waveforms by modality and condition, as a function of ASD and Conversion status. Figures 2A,B show z-score corrected P300 amplitudes for auditory and visual modalities, respectively. The estimated regression parameters are summarized in Table 3. A main effect of P300 amplitude predicting conversion status was significant in the visual modality (P3a: *p* = 0.004; P3b: *p* = 0.004) and in the auditory modality for P3b (*p* = 0.045), but not P3a (*p* = 0.78).The ASD × P300 Amplitude interaction significantly predicted conversion status in models of both Novel (P3a: *p* = 0.001) and Target (P3b: *p* = 0.001) stimuli in the visual modality, and for Target stimuli (P3b) in the auditory modality (*p* = 0.039), but not for auditory novel stimuli (P3a: *p* = 0.80).

**Table 3:** Binomial logistic regression summary with bootstrapping of oddball ERP data.

**Model**	**B**	**SE**	**OR**	***p***
**Visual – Novel**				
P3a Amplitude	1.47	0.78	4.36	0.004
Amplitude × ASD Diagnosis	−1.80	0.80	–	0.001
**Visual – Target**				
P3b Amplitude	1.56	0.72	4.73	0.004
Amplitude × ASD Diagnosis	−2.16	0.75	–	0.001
**Auditory – Novel**				
P3a Amplitude	−0.12	0.47	0.89	0.78
Amplitude × ASD Diagnosis	0.12	0.48	–	0.80
**Auditory – Target**				
P3b Amplitude	2.98	1.54	19.70	0.045
Amplitude × ASD Diagnosis	−3.37	1.56	–	0.039

**Figure 3:**
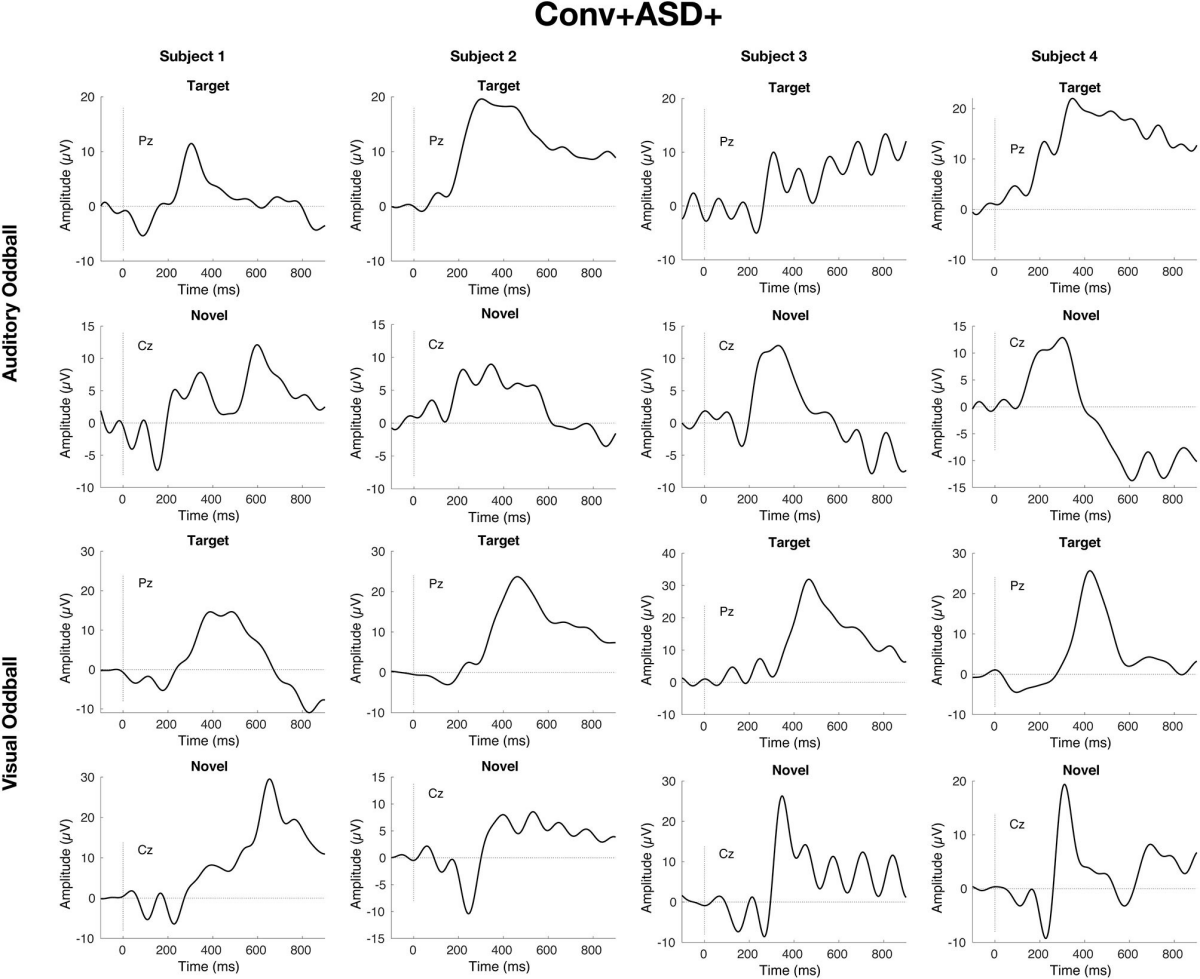
Individual waveforms for *n* = 4 CHR participants with ASD history who convert to psychosis within 2 years of their baseline visit and EEG.

Simple slopes analyses indicated that, within the ASD+ group, more enhanced P300 amplitudes (relative to the TD sample against which they were z-scored) were significantly associated with conversion to psychosis for both auditory and visual target stimuli, as well as for visual novel stimuli (Auditory P3b: OR = 16.72, β = 2.82, SE = 1.49, *p* = 0.011; Visual P3b: OR = 7.80, β = 2.05, SE = 1.21, *p* = 0.025; Visual P3a: OR = 4.47, β = 1.50, SE = 0.83, *p* = 0.008). There was no association between Auditory P3a amplitude and psychosis conversion in the ASD+ group (*p* = 0.93). See Figure 3 for individual waveforms by condition from all four Conv+/ASD+ participants. These findings contrast with the ASD– CHR subset, wherein P300 enhancements were significantly associated with decreased risk of conversion to psychosis (Auditory P3b: OR = 0.67, β = −0.39, SE = 0.16, *p* = 0.006; Visual P3a: OR = 0.72, β = −0.32, SE = 0.13, *p* = 0.015; Visual P3b: OR = 0.55, β = −0.60, SE = 0.17, *p* = 0.001), consistent with previous literatures wherein attenuated P300 amplitudes typically associate with conversion to psychosis.

## Discussion

In this paper, we present exploratory analyses of the utility of EEG markers for predicting conversion to psychosis in a unique, albeit small, sample of individuals with ASD at clinical high risk for psychosis, followed longitudinally for two years. We find that P300 amplitude profiles to visual target and novel and auditory target stimuli in CHR patients differentially predict conversion to psychosis as a function of ASD status. In the general CHR population, previous literature shows that reduced P300, and particularly P3b response to behaviorally-relevant auditory stimuli, is both characteristic of the group as a whole and predictive of later conversion to psychosis. Here, we find preliminary evidence that history of ASD diagnosis moderates this relationship. In particular, enhanced – rather than attenuated - P300 response to visual and target stimuli appears to be a unique profile associated with conversion to psychosis among CHR individuals with ASD history. Whereas, intact or enhanced P300 response is typically a positive prognostic marker in the CHR literature, we show that, for every one standard deviation increase in P300 amplitude above the mean in healthy controls, CHR individuals with ASD history have between 4 and 16 times greater chance of developing psychosis. Such pattern was not characteristic of either CHR individuals without ASD, or CHR individuals with ASD who did not convert to psychosis. Moreover, the observed odds ratios for P300 predicting conversion to psychosis in ASD are strikingly large compared to those often seen in studies examining predictors of psychosis in broader CHR groups. Neither accuracy nor reaction time during task performance differed between CHR patients with ASD who converted to psychosis and any of the other study groups, and the amount of data retained for analysis also did differ by group. These factors contribute to early confidence that observed differences in ERP response likely reflect true differences in brain response, rather than being artifactual as a function of differences in behavior response patterns or data quality.

Our oddball task findings in CHR patients without ASD align with previous work showing that P300 amplitude is reduced in psychosis (18, 19), in CHR (29, 30, 73), and in those with CHR who convert to psychosis (32, 33, 60, 74). In patients with CHR who have a prior ASD history but do not convert to psychosis, we also see P300 reductions that are consistent with both the broader CHR group and the general literature on P300 in ASD without psychosis (61, 75). Indeed, the pattern of enhanced P300 to visual and target stimuli appears to be unique to those with CHR and ASD whose illness trajectory results in full-blown psychosis within 2 years. It may reflect allocation of an aberrantly large degree of attention to sensory input, in the visual domain, regardless of behavioral relevance of stimuli, as well as when sensory input is behaviorally relevant, regardless of sensory domain. Of note, as accuracy of responding to target stimuli did not differ among participants as a function of ASD or conversion status, post-attention decision making steps may still function similarly, at least in the context of a simple detection task, despite differential attentional allocation at the neural level.

These results provide initial evidence suggesting that ASD status may be important to account for when evaluating neural markers that may predict later transition to psychosis in CHR individuals. This finding is interesting in light of the fact that clinical predictors of conversion to psychosis do not seem distinct in CHR individuals with ASD vs. those without (17), suggesting additive information from the neural data. Based on prior literature (32), more attenuated P300 amplitude in CHR individuals ought to raise greater concern about future conversion to psychosis. Thus, with such literature as background and without knowledge of prior ASD status, discovering *enhanced* P300 amplitude to oddball stimuli in a CHR individual might be cause for optimism about prognosis and recovery. If borne out in larger studies, our results suggest that knowing the ASD history of these individuals may therefore be of import: only if one know the individual's prior ASD diagnosis can one make the more nuanced interpretation, raising concern about conversion as a function of the P300 enhancement. Combined with clinical and demographic indicators of risk for conversion to psychosis, this information from EEG could in turn contribute to more accurate predictions about disease trajectory.

Study findings are of course limited by our small sample of CHR individuals with ASD, particularly for those who converted to psychosis. However, the large odds ratios we uncovered support the import of this hypothesis-generating work. In addition, our sample consists only of help-seeking individuals, who are plausibly not entirely representative of the broader population of those with ASD and psychotic-like symptoms. Finally, CHR itself is a broad category, and the range of concerning symptoms expressed at baseline was likely heterogenous both within our ASD subset and within the broader CHR group. Due to our small sample size, we did not look at individual clinical symptom associations, but baseline symptoms did not differ among those with or without ASD, or who did or did not convert to psychosis. Despite study limitations, the striking dissociation among groups that we discovered provides an exemplar of why this line of work is critically important, as our findings would be entirely masked were ASD status not considered. Samples of ASD individuals with CHR symptoms followed longitudinally are exceedingly rare to date, making our findings important, even if preliminary. Future studies in larger samples of CHR individuals with ASD and comparing to non-CHR ASD are needed in order to validate our preliminary findings and ensure they are not spurious. Should they replicate in larger samples, our results could mean new insight into prevention and intervention in patients presenting to CHR clinics with ASD history, and for those presenting to ASD clinics with early signs of psychosis.

## Data Availability Statement

The data analyzed in this study is subject to the following licenses/restrictions: data belongs to the NAPLS2 consortium and may be available upon request. Requests to access these datasets should be directed to daniel.mathalon@ucsf.edu.

## Ethics Statement

Study procedures were reviewed by and approved across all eight sites of the NAPLS2 consortium. Written informed consent to participate in this study was provided by the participant, or by the participant's legal guardian for those under 18 years of age.

## Author Contributions

JF-F, EV, PB, AB, GL, MN, KC, TM, DP, LS, SW, TC, and DM: concept and design. JF-F, SG, BR, HH, PB, RC, ED, JJ, GL, MN, JA, CB, KC, BC, LS, WS, MT, EW, SW, TC, and DM: acquisition, analysis, or interpretation of data. JF-F, SG, BR, HH, and DM: drafting of the manuscript. JF-F, SG, BR, EV, HH, PB, AB, RC, ED, JJ, GL, MN, JA, CB, KC, BC, TM, DP, WS, MT, EW, SW, TC, and DM: critical revision of the manuscript for important intellectual content. JF-F, SG, BR, and DM: statistical analysis. JA, CB, KC, BC, DP, LS, EW, TC, and DM: obtained funding. HH, BR, PB, RC, ED, GL, MN, JA, CB, KC, BC, LS, WS, EW, TC, and DM: administrative, technical, or material support. All authors contributed to the article and approved the submitted version.

## Conflict of Interest

The authors declare that the research was conducted in the absence of any commercial or financial relationships that could be construed as a potential conflict of interest.
